# Does Hospitalization Change the Perception of COVID-19 Vaccines among Unvaccinated Patients?

**DOI:** 10.3390/vaccines10030476

**Published:** 2022-03-19

**Authors:** Dorota Zarębska-Michaluk, Piotr Rzymski, Anna Moniuszko-Malinowska, Michał Brzdęk, Diana Martonik, Marta Rorat, Jakub Wielgat, Krzysztof Kłos, Witold Musierowicz, Piotr Wasilewski, Włodzimierz Mazur, Barbara Oczko-Grzesik, Monika Bociąga-Jasik, Justyna Kowalska, Robert Flisiak

**Affiliations:** 1 Department of Infectious Diseases, Jan Kochanowski University, 25-317 Kielce, Poland; michal.brzdek@gmail.com; 2 Department of Environmental Medicine, Poznan University of Medical Sciences, 60-806 Poznan, Poland; rzymskipiotr@ump.edu.pl; 3 Integrated Science Association (ISA), Universal Scientific Education and Research Network (USERN), 60-806 Poznan, Poland; 4 Department of Infectious Diseases and Neuroinfections, Medical University of Bialystok, 15-089 Bialystok, Poland; annamoniuszko@op.pl; 5 Department of Infectious Diseases and Hepatology, Medical University of Bialystok, 15-089 Bialystok, Poland; diana.martonik@umb.edu.pl (D.M.); robert.flisiak1@gmail.com (R.F.); 6 Department of Forensic Medicine, Wrocław Medical University, 50-149 Wroclaw, Poland; marta.rorat@gmail.com; 7 First Infectious Diseases Ward, Gromkowski Regional Specialist Hospital in Wroclaw, 50-149 Wroclaw, Poland; 8 Department of Infectious Diseases and Hepatology, Medical University of Lodz, 90-549 Lodz, Poland; jakubwielgat@gmail.com; 9 Department of Infectious Diseases and Allergology, Military Institute of Medicine, 04-349 Warsaw, Poland; kklos@wim.mil.pl; 10 Department of Infectious Diseases and Hepatology, Faculty of Medicine, Collegium Medicum in Bydgoszcz, Nicolaus Copernicus University, 87-100 Torun, Poland; witold.musierowicz@cm.umk.pl; 11 4th Department, Hospital for Infectious Diseases in Warsaw, 01-201 Warsaw, Poland; p.wasilewski@onet.eu; 12 Clinical Department of Infectious Diseases in Chorzow, Medical University of Silesia, 40-055 Katowice, Poland; wlodek.maz@gmail.com; 13 Department of Infectious Diseases and Hepatology, Medical University of Silesia, 40-055 Katowice, Poland; bgrzesik@hoga.pl; 14 Department of Infectious and Tropical Diseases, Faculty of Medicine, Jagiellonian University Medical College, 31-007 Krakow, Poland; monika.bociagajasik@gmail.com; 15 Department of Adults’ Infectious Diseases, Medical University of Warsaw, 02-091 Warsaw, Poland; jdkowalska@gmail.com

**Keywords:** vaccine hesitancy, pandemic, SARS-CoV-2, COVID-19, misinformation

## Abstract

The COVID-19 vaccination has been the subject of unprecedented misinformation, false news, and public concerns. This study presents a unique analysis comprising persons who were not vaccinated and became ill. It investigates reasons for not vaccinating and evaluates how the personal experience of COVID-19 affected further attitudes and decisions related to health. The study included 730 consecutive unvaccinated patients hospitalized in 12 centers in Poland during the autumn 2021 pandemic wave. The most frequent reason behind the refusal to receive the vaccine was concern over the adverse effects, disbelief that the vaccine was sufficiently tested, and one’s conviction that COVID-19 will not affect a patient. Online information, friends, spouse, children/grandchildren, and other family members were most often the source of discouragement from vaccination. Most individuals regretted their decision not to receive a vaccine (66.0%), declared to promote COVID-19 vaccination after discharge (64.0%), and to receive a COVID-19 vaccine in the time recommended for convalescents (69.5%). Individuals expressing no regrets of vaccine refusal more frequently revealed conspiracy beliefs. The study shows that personal experience with severe COVID-19 can influence the perception of vaccination, but approximately one-third of unvaccinated hospitalized patients still appear to express vaccine hesitancy.

## 1. Introduction

Vaccination campaigns represent one of the essential methods to fight the COVID-19 pandemic. The priority of vaccination is to decrease the clinical severity, need for hospitalization, and risk of death. The prevention of SARS-CoV-2 infection, whether symptomatic or asymptomatic, is considered as a secondary goal, yet it is achieved to some extent despite the emergence of novel, more transmissible variants adapted better to evade humoral immunity [1,2,3]. Since unvaccinated individuals remain the main drivers of viral spread in the population and support the mutation dynamics of SARS-CoV-2 [4], the primary vaccination of unvaccinated individuals remains the priority [5]. Unfortunately, vaccination campaigns have been targeted by anti-science movements perpetuated by unprecedented misinformation. This misinformation started long before the vaccines were authorized and was rooted in the belief that the pandemic was deliberately induced for profits from selling vaccines [6]. It has subsequently increased vaccine hesitancy and lower vaccination rates in selected populations, such as the Polish. Despite various initiatives to communicate vaccine science to the general public [7], accumulating evidence that COVID-19 vaccination is saving lives and can decrease the overwhelming of healthcare systems [8,9,10,11], only 55.5% of the Polish population (including 64% adults) was vaccinated at the end of 2021. Studies have shown that apart from beliefs in conspiracy theories, the main reasons for vaccine hesitancy included fear of acute side effects and feeling physically fit and healthy [12]. Notably, between September and December, the share of fully vaccinated individuals in Poland only increased from 49.6% to 56.2% [13], even though during the same period, over 21 thousand deaths due to COVID-19 were recorded [14].

There are many determinants of COVID-19 vaccine acceptance, including gender, trust in medical, scientific experts, and public health authorities, worry about severe disease, and family COVID-19 experience [15,16]. The question remains whether hospitalization of unvaccinated patients could influence their further vaccine acceptance and perception. It can be hypothesized that the severity and clinical course of the disease may have a significant effect in this regard. On the other hand, individuals emotionally attached to conspiracy theories related to the COVID-19 pandemic and vaccines may struggle to abandon their beliefs despite the experience of severe disease. For example, some individuals living with HIV/AIDS may reveal a strong AIDS denialism: they may believe there is no proof that HIV causes AIDS and that HIV treatments do more harm than good [17]. Moreover, a flood of diverse misinformation on the COVID-19 vaccines on an unprecedented scale has caused concerns over vaccine safety and efficacy in individuals who may not otherwise be prone to conspiracy beliefs. Although many papers assessed the reasons behind COVID-19 vaccine refusal, no studies have so far investigated them in unvaccinated patients hospitalized with severe COVID-19 or whether this experience had any effect on their views on vaccination. 

The present study aimed to analyze the reasons for the lack of vaccination in Polish patients hospitalized with COVID-19 during the pandemic between November and December 2021. The sources of discouragement from vaccination were also identified. Moreover, the study assessed whether severe disease experience could potentially change the future perception of the COVID-19 vaccines in unvaccinated patients.

## 2. Materials and Methods

### 2.1. Design and Data Collection

We conducted a real-world multicenter observational study including consecutive unvaccinated adult patients with COVID-19 hospitalized between 1 November 2021 and 15 December 2021 in 12 infectious diseases departments in Poland. During this period, there were no shortages in COVID-19 vaccine availability. The vaccine coverage in the entire population exceeded 50% in mid-September 2021 and increased only by 4.7% until the end of this study, indicating a high vaccine hesitancy in Poland. 

Data were retrieved retrospectively from the medical documentation. Patient consent was waived due to the retrospective design of the study. The criteria for inclusion included being unvaccinated and being able to answer questions from a medical interview consciously.

The demographic data included age, gender, level of education, place of residence (urban/rural), occupation status (professionally active/inactive), and whether an individual was a healthcare worker. Patients were asked about their reasons for vaccine refusal and whether this decision was influenced by other persons, institutions, or opinions found in media or the internet. Furthermore, each patient was asked whether they (i) regret the decision not to receive a vaccine, (ii) intend to promote vaccination when discharged from the hospital, and (iii) intend to receive COVID-19 vaccine when discharged from the hospital. The initial information was obtained as a part of a routine medical and epidemiological interview collected upon admission of a patient with COVID-19 to the hospital according to the standpoint 24 of the Medical Council at the Prime Minister of Poland of 15 September 2021 (https://www.gov.pl/web/koronawirus/rada-medyczna, accessed on 1 March 2022). The information on the attitude to vaccinations after hospitalization was collected from patients who survived on the day of discharge as a part of a routine hospital procedure. In addition, data on the most advanced condition of the patient during hospitalization were collected from the medical records. 

### 2.2. Statistical Analysis

Statistica v.13.1 (StatSoft Inc., Tulsa, OK, USA) was used for data analysis. Because age did not meet the assumption of Gaussian distribution, a non-parametric Mann–Whitney U test was applied to test its effect. The differences in frequencies of given answers were assessed with Pearson’s χ^2^ test. A *p*-value < 0.05 was considered statistically significant.

## 3. Results

### 3.1. Demographic Characteristics

Data from 730 consecutive unvaccinated patients hospitalized due to COVID-19 in Poland were retrospectively collected and analyzed. The demographic breakdown of the studied group was summarized in Table 1. The majority of individuals were aged ≥ 60 years (52.2%), lived in urban areas (81.1%), and had secondary (32.2%) or tertiary education (29%). The share of women and men and professionally active and inactive individuals were nearly equal. Overall, 23 patients (3.2%) were healthcare workers, including medical doctors, midwives, nurses, dental assistants, radiology technicians, pharmacologists, and sanitary staff. Most hospitalized patients required oxygen therapy, predominantly in the form of low-oxygen supplementation. The death rate in the studied group was 8.6% (Table 1). 

### 3.2. Reasons for COVID-19 Vaccine Refusal

**Figure 1:**
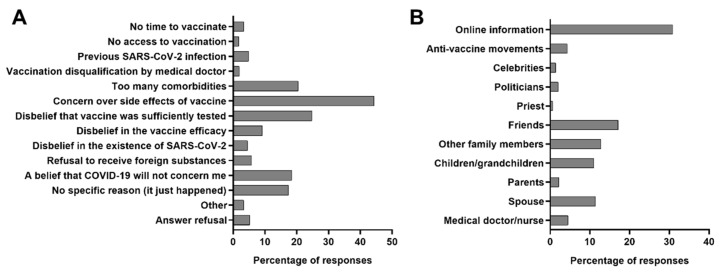
The frequency of reasons behind the refusal to receive the vaccine (**A**) in the whole studied group of patients hospitalized due to COVID-19 in Poland (*n* = 730), and (**B**) those whose decisions were made under the influence of opinions of other people or organizations (*n* = 377).

Studied patients declared various reasons for refusal to receive the COVID-19 vaccine. The most frequent included: concern about the adverse effects of the vaccine (44.2%), belief that the vaccine has not been sufficiently tested (24.8%), patients’ belief that they have too many comorbidities to receive a vaccine (20.4%), and one’s conviction that COVID-19 will not affect them (18.5%) (Figure 1A). Approximately half of the patients (51.6%) declared that their decision not to receive the COVID-19 vaccine was made under the influence of the opinion of other people or organizations, most often due to information found on the internet (30.8%), friends (17.1%), spouse (11.4%), children or grandchildren (11.0%), and other family members (12.8%) (Figure 1B). 

Only one patient admitted to being a part of the anti-vaccine organization. Healthcare workers (medical workers and nurses) discouraged only 4.5% of patients. Effective discouragement by more than one source was reported by 28.9% of patients. 

### 3.3. Regrets of COVID-19 Vaccine Refusal

**Figure 2:**
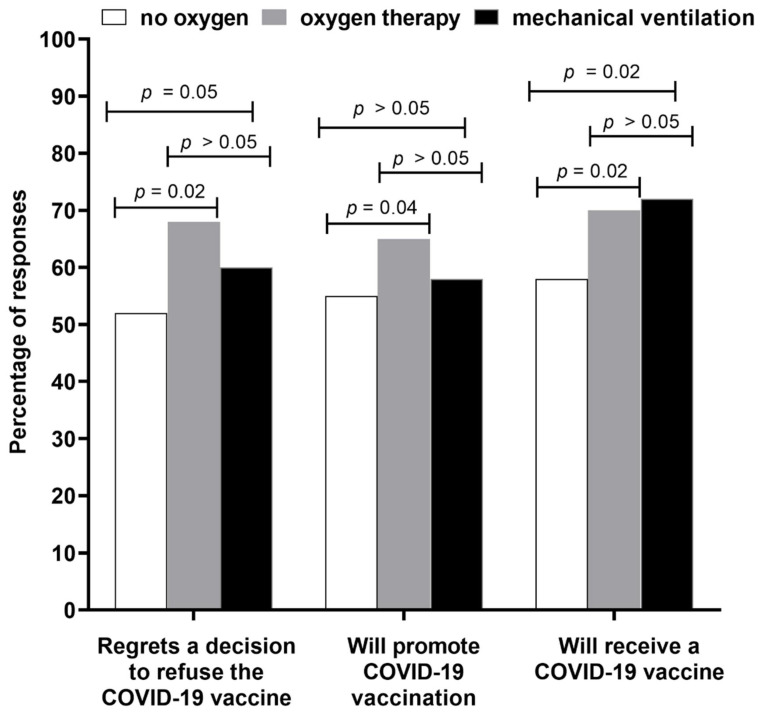
The frequency of various declarations made by unvaccinated hospitalized patients (*n* = 730) in relation to the clinical severity of COVID-19.

The majority of patients expressed regretting their decision not to receive a vaccine (66.0%), 14.7% did not regret it, while 18.3% were unsure about their feelings. No difference in this regard was noted between men and women, those inhabiting urban and rural areas, and professionally active and inactive (*p* > 0.05 in all cases, Pearson’s χ^2^ test); age also was not a differentiating factor (*p* > 0.05, Mann–Whitney U test). Patients regretting this decision the least often had tertiary education (58.6%) followed by primary (65.6%), secondary (67.4%), and vocational (73.1%) (*p* = 0.02, Pearson’s χ^2^ test). Only 54.5% of healthcare workers expressed such regret. Patients who did not require oxygen therapy or mechanical ventilation revealed the lowest frequency of regretting the refusal of the COVID-19 vaccine (Figure 2). 

Patients not regretting the vaccine refusal were more frequently indicating the following reasons: disbelief that vaccine was sufficiently tested (77.7% vs. 53.6%, *p* < 0.0001, Pearson’s χ^2^ test), doubt in vaccine efficacy (78.0% vs. 40.7%, *p* < 0.0001, Pearson’s χ^2^ test), disbelief in the existence of SARS-CoV-2 (74.2% vs. 13.6%, *p* < 0.005, Pearson’s χ^2^ test), and refusal to receive foreign substances to the organism (68.4% vs. 12.4%, *p* < 0.0001, Pearson’s χ^2^ test). No significant differences in frequency of responses were found for other reasons presented in Figure 1A. 

### 3.4. Attitudes toward COVID-19 Vaccine after Severe Disease Experience

Most of the studied patients (64.0%) declared to promote the COVID-19 vaccination when discharged from the hospital (14.6% declared against, while 20.4% were unsure) and to receive their COVID-19 vaccine (69.5%) in the time recommended for convalescents (10.1% declared no willingness to receive it, while 20.4% were unsure). Gender, place of residence, and occupation status did not differentiate attitude toward these two aspects (*p* > 0.05 in all cases, Pearson’s χ^2^ test), similarly to age (*p* > 0.05 in both cases, Mann–Whitney U test). Individuals regretting their decision to refuse the COVID-19 vaccine were more frequently willing to promote the vaccinations (86.3% vs. 17.7%, *p* < 0.0001, Pearson’s χ^2^ test) and to receive COVID-19 vaccine (71.8% vs. 60.4%, *p* < 0.01, Pearson’s χ^2^ test).

Among healthcare workers, only 59.1% declared to promote COVID-19 vaccination, while 72.7% expressed a will to receive the COVID-19 vaccine. Patients with tertiary education were the least often willing to accept a COVID-19 vaccine after hospital discharge (59.6%) compared to those with vocational (71.2%), secondary (72.5%), and primary (75.0%) education levels (*p* = 0.04, Pearson’s χ^2^ test). 

Similarly, patients with less severe COVID-19 (not requiring oxygen therapy or mechanical ventilation) were also less frequently willing to receive the COVID-19 vaccine when compared to those on low- and high-oxygen supplementation as well as mechanical ventilation. 

Individuals requiring mechanical ventilation most often regretted the decision not to receive a vaccine, declared to promote COVID-19 vaccination after being discharged from the hospital and to receive COVID-19 vaccine as recommended. 

Significant differences between patients not requiring oxygen supplementation and those using oxygen therapy and mechanical ventilation were found, regarding regrets in their decision to refuse the COVID-19 vaccine, intent to promote the vaccine, and intent to receive vaccination after discharge, while between patients requiring oxygen therapy and those using mechanical ventilation a statistically significant difference was documented only for a declaration to receive a vaccine as recommended for convalescents (Figure 2).

## 4. Discussion

The present analysis presents the perspective on COVID-19 vaccines among unvaccinated patients hospitalized due to COVID-19 during the autumn 2021 pandemic wave in Poland, during which vaccination was freely available to everyone willing to receive it. Although there are numerous studies available assessing the level of COVID-19 vaccine acceptance and analyzing the reasons for their refusal in different populations and demographical groups [18,19], the nature of our research was unique—it included unvaccinated patients suffering from severe COVID-19 and requiring hospitalizations. As far as we are concerned, this is the first study to investigate the reasons behind vaccine refusal in unvaccinated COVID-19 hospitalized patients and assess whether hospitalization has influenced the patients’ perspectives and views toward the vaccinations. 

No universal profile of unvaccinated individuals was found, while various reasons behind the vaccine refusal were reported. Unsurprisingly, the concerns over adverse effects were the most frequent motive behind vaccine refusal (44%)—this finding is in line with many other investigations conducted in various populations [20,21,22]. Such a reason can be considered rationale as patients are entitled to have their fears over the vaccine, particularly in populations in which vaccinations of adults are rare. This is the case in Poland, where the influenza vaccination coverage is very low (<5%) [23]. In contrast, the second most frequently indicated reason for COVID-19 vaccine refusal, i.e., that vaccines had not been sufficiently tested, reported by one-fourth of studied patients, should be regarded as unjustified in the light of the available scientific data. Interestingly, this reason was more frequently indicated by patients with tertiary education (35%). This is because numerous previous studies have shown that low vaccination is strongly tied to vaccine hesitancy, including in the context of COVID-19 vaccines [24,25,26]. However, vaccine skepticism is a multifaceted phenomenon and can sometimes also be seen in better-educated groups, e.g., due to critical-reflexive repertoire that enables questioning of what is considered to be scientific consensus [27]. Our study also highlights that in the case of COVID-19 vaccinations, hesitancy is not only present in less-educated individuals but may encompass various groups. This should be taken into account in shaping the vaccination promotion campaigns. It is important to communicate the results of clinical trials and epidemiological studies to the public while stressing that they also come with limitations and unknowns instead of expressing extensive optimism. This is particularly relevant in the case of vaccines developed and authorized at an unprecedented pace during the pandemic. 

As many as 52% of studied patients admitted that their decisions were influenced by others’ opinions or organizations, indicating information from the internet as the most common source. The anti-vaccine content published online, especially on social media platforms, has been a prominent issue throughout the pandemic [28]. The myths, conspiracy theories, misinformation, and pseudoscientific misconception about COVID-19 and COVID-19 vaccines posted online are rapidly reaching millions of recipients globally and may strongly influence individuals’ decision-making [29,30]. Some cross-sectional analyses documented that mainstream media (TV, newspapers) and social media may negatively affect a subject’s vaccine intent due to the spread of false information [31,32]. 

A small percentage of patients (4.5%) in the analyzed group indicated healthcare workers as the source of information based on which the decision not to vaccinate was made. The share of this professional group in the overall analyzed population of unvaccinated hospitalized patients was also small (3.2%). Similar to other countries, Polish healthcare workers were prioritized in access to vaccination [33]. They represent the occupational group with the highest vaccination coverage, although some hesitancy toward COVID-19 vaccines is still present [34,35,36,37]. As also found, unvaccinated healthcare workers hospitalized due to COVID-19 regretted their decision not to vaccinate less often, and 43% were unwilling to promote vaccination or were unsure about it despite their experience with severe COVID-19. Since they represent one of the most trusted sources of vaccine-related data and play a role as vaccination advisors, their attitudes are highly influential on patients’ decisions. While the impact of family members and friends on vaccine refusal was significant in the studied group, the influence of celebrities, politicians, and priests turned out to be marginal.

The added value of the present research was the assessment of the role of personal experience in shaping the perception and decisions related to health. Most hospitalized patients regretted their decision to refuse the COVID-19 vaccine, declared to support COVID-19 vaccination after being discharged, and expressed a will to receive their COVID-19 vaccine in the time recommended for convalescents. Importantly, severe COVID-19 experience did not convince approximately one-third of studied patients to receive vaccination. As evidenced, those who did not regret their refusal of the COVID-19 vaccine were more likely to support their decision with claims related to conspiracy theories, such as disbelief in the existence of SARS-CoV-2. This highlights the importance of misinformation on COVID-19 vaccines on some individuals who are emotionally attached to their beliefs and not prone to abandon them despite contradictory personal experiences. The pre-pandemic evidence shows that this may mainly concern those with anxiety traits, perceiving that society is under threat and that the situation lacks control [38,39,40]. As also found, unvaccinated healthcare workers hospitalized due to COVID-19 regretted their decision not to vaccinate less often, and 26.1% were unwilling to promote vaccination despite their experience with severe COVID-19. Considering that most healthcare workers are vaccinated in Poland, this may be related to the active polarization between the vaccinated and unvaccinated, representing the same professional group [41]. 

The study has some limitations. Social attitudes may influence patients’ responses, therefore it cannot be entirely ruled out that some of them provided the answers expected by the medical doctor on whom their health and life depended. Moreover, the willingness of patients to receive vaccination after hospitalization was not verified later, and it is possible that some of the patients who declared a change in perception of COVID-19 vaccination ultimately did not receive the vaccine. The study’s strength is the collection of data from a real-world, diverse population representative of routine practice. Moreover, the data originated from a specific, unique group of unvaccinated patients hospitalized with COVID-19, not from the general population. In addition, the study comprised patients with various degrees of COVID-19 severity, including seriously ill individuals requiring intensive care treatment and mechanical ventilation in the further course of the disease. 

## 5. Conclusions

Our study found that despite wide access to vaccines some patients may still decide to risk their health and lives by not vaccinating. Suffering from severe COVID-19 appeared to change most unvaccinated patients’ attitudes toward vaccinations. However, one-third of unvaccinated hospitalized patients still expressed vaccine hesitancy. Its highest levels were seen in individuals who refused the COVID-19 vaccine based on conspiracy theories and false information. Unvaccinated individuals who suffered less severe disease and did not require oxygen supplementation were also less convinced of the benefits of COVID-19 vaccines. The study adds to the evidence that convincing some individuals to receive COVID-19 vaccination may be a challenging task, particularly in groups exposed to misinformation and conspiracy theories. 

## Figures and Tables

**Table 1:** Demographic characteristics of unvaccinated Polish patients hospitalized due to COVID-19 included in the study (*n* = 730).

Parameter	Value
**Gender,** women/men, *n* (%)	374 (51.2)/356 (48.8)
**Age** (years) mean ± SD; min-max	58.9 ± 16.3; 19–98
**Place of residence**, urban/rural, *n* (%)	592 (81.1)/138 (18.9)
**Level of education**, *n* (%)	
Primary education	104 (14.3)
Vocational education	176 (24.1)
Secondary education	235 (32.2)
Tertiary education	212 (29)
No data	3 (0.4)
**Occupational status**, *n* (%)	
Professionally active/inactive	355 (48.6)/375 (51.4)
**Healthcare worker**, *n* (%)	23 (3.2)
**Clinical severity**	
No oxygen therapy needed, *n* (%)	74 (10.1)
Low-flow oxygen therapy needed, *n* (%)	448 (61.3)
High-flow oxygen therapy needed, *n* (%)	122 (16.7)
Mechanical ventilation or ECMO, *n* (%)	86 (11.9)
**The fatal outcome**, *n* (%)	63 (8.6)

ECMO, Extra Corporeal Membrane Oxygenation.

## Data Availability

Data supporting reported results can be provided upon request from the corresponding author.
